# A New Use for Hospital Gardens: The Cultivation of Medicinal Herbs

**Published:** 1916-08-26

**Authors:** 


### A NEW USE FOR HOSPITAL GARDENS.

### The Cultivation of Medicinal Herbs.

In a brief note in The Hospital of April 22 (p. 77) it was suggested that in view of the great and constantly increasing scarcity of medicinal plants it would be a wise policy to utilise land adjoining hospitals for the purpose of growing herbs used in medicine. As this is the season of the year which is the busiest for garden operations, it would seem appropriate to deal with this subject in greater detail, to suggest plants which might with profit be grown, to give some hints on cultivating them, and indicate where further information may be obtained.

### Why Drug-yielding Plants are Scarce.

It is by this time common knowledge that until the outbreak of war this country was mainly dependent upon Central Europe for its supplies of some of the most commonly prescribed vegetable drugs. The consequence of cutting off this source of supply has been to make it difficult to procure even at extravagant prices stocks of various roots, flowers, herbs, and leaves that are in everyday use in hospital practice. Belladonna, aconite, foxglove, valerian, and henbane, to mention only a few, could probably be produced in our own country in sufficient quantities to supply our own needs and to have some left over for export. All of them are grown in Great Britain at the present time, but on a scale that is insufficient to meet the demand.

Now a hospital garden is most suitable land for cultivating these and other plants for a very special reason—namely, that all hospitals, or, at any rate, all large hospitals are provided with facilities for drying herbs or for making extracts from fresh herbs as soon as they are collected. Further, a hospital has at its service, in the person of the pharmacist, someone who has been trained to identify herbs and has studied botany—especially the botany of drug-yielding plants. It is open to question whether it is altogether desirable to encourage amateurs to grow such plants as belladonna and aconite, but a medicinal herb garden cultivated under the supervision of one who has a knowledge of the physical characteristics and therapeutic properties of plants is desirable and appropriate. Another advantage of associating such a garden with a hospital pharmaceutical laboratory is that experiments could be undertaken with the object of studying the conditions under which the alkaloidal content of certain poisonous plants is subject to variation. A further advantage is that galenical preparations could be produced at least equal in quality to those bought in the open market, and probably at a smaller cost to the finances of the institution.

It is a curious fact that while most of the few medicinal plants that are still cultivated in this country are esteemed for their quality all over the world, the quantity produced is so small. That the climate is eminently suitable for certain plants is obvious, and in many districts the soil is the most desirable, while in others by proper treatment and manuring it could be made suitable. At one time, in fact, the cultivation of medicinal plants was a flourishing English industry, and it is common knowledge that in the gardens adjoining monastic establishments medicinal herbs were grown on a systematic plan. The Board of Agriculture having recognised the desirability of increasing our supplies of much-needed drugs, has recently issued a revised leaflet (No. 288), a copy of which should be in the hands of all hospital gardeners and hospital pharmacists. Therein it is shown what drugs are most needed, what plants can be cultivated in this country, and what are the appropriate conditions of cultivation and harvesting.

### Plants Worth Growing.

Special instructions are given regarding aconite, belladonna, chamomile, dandelion, foxglove, dill, fennel, henbane, white poppy, thorn apple, valerian, male fern, and colchicum; while general information is supplied as to other plants. *Aconite* prefers a soil slightly retentive of moisture; it is best propagated from the smaller roots, which develop at the sides of the old roots. If it is grown from the seed great care must be exercised in ensuring that the right kind is obtained, as there are many varieties of aconite. *Belladonna* prefers a chalky substratum, but will grow on most soils. Seeds are practically unobtainable at the present time. *Chamomile* prefers a dry sandy loam. The usual method of propagation is by division of the old plant into ten or twelve parts. It can also be grown from seed. *Dandelion* grows in almost any soil, about 4 lb. of seeds per acre are drilled in rows a foot apart, and the roots are dug the second year in the autumn. *Dill* is cultivated in a similar way to spring oats. *Fennel* yields best on rich soil and likes plenty of sun. *Henbane*, like belladonna, will grow on most soils; seeds are very difficult to obtain, regular herb farmers having required all they could get for their own farms. *White Poppy* prefers rich, moist soil with plenty of sun; about 1 lb. of seed per acre is drilled in rows a foot apart. *Stramonium* grows well in sunny situations; the seeds (10 lb. to 15 lb. per acre) are drilled in rows two feet apart. *Valerian* is cultivated from wild plants, and planting is usually done on land treated with farmyard manure.

The authority responsible for selecting what medicinal plants should be grown in a hospital garden would have little difficulty in determining which to avoid and which to select for cultivation. Some plants as, for instance, foxglove, colchicum, and dandelion, could be left to those having large areas of land at their disposal, or, as the plants grow wild, they could be collected on some organised plan. Belladonna, aconite, and henbane, however, are among the plants which might with advantage be selected for the hospital garden.

